# Widespread contamination of SARS‐CoV‐2 on highly touched surfaces in Brazil during the second wave of the COVID‐19 pandemic

**DOI:** 10.1111/1462-2920.15855

**Published:** 2022-01-20

**Authors:** Severino Jefferson Ribeiro da Silva, Jessica Catarine Frutuoso do Nascimento, Wendell Palôma Maria dos Santos Reis, Caroline Targino Alves da Silva, Poliana Gomes da Silva, Renata Pessôa Germano Mendes, Allyson Andrade Mendonça, Bárbara Nazly Rodrigues Santos, Jurandy Júnior Ferraz de Magalhães, Alain Kohl, Lindomar Pena

**Affiliations:** ^1^ Laboratory of Virology and Experimental Therapy (LAVITE), Department of Virology Aggeu Magalhães Institute (IAM), Oswaldo Cruz Foundation (Fiocruz) Recife Pernambuco 50670‐420 Brazil; ^2^ Department of Virology Pernambuco State Central Laboratory (LACEN/PE) Recife Pernambuco Brazil; ^3^ University of Pernambuco (UPE), Serra Talhada Campus Serra Talhada Pernambuco Brazil; ^4^ MRC‐University of Glasgow Centre for Virus Research Glasgow G61 1QH UK

## Abstract

Although SARS‐CoV‐2 surface contamination has been investigated in health care settings, little is known about the SARS‐CoV‐2 surface contamination in public urban areas, particularly in tropical countries. Here, we investigated the presence of SARS‐CoV‐2 on high‐touch surfaces in a large city in Brazil, one of the most affected countries by the COVID‐19 pandemic in the world. A total of 400 surface samples were collected in February 2021 in the City of Recife, Northeastern Brazil. A total of 97 samples (24.2%) tested positive for SARS‐CoV‐2 by RT‐qPCR using the CDC‐USA protocol. All the collection sites, except one (18/19, 94.7%) had at least one environmental surface sample contaminated. SARS‐CoV‐2 positivity was higher in public transport terminals (47/84, 55.9%), followed by health care units (26/84, 30.9%), beach areas (4/21, 19.0%), public parks (14/105, 13.3%), supply centre (2/21, 9.5%), and public markets (4/85, 4.7%). Toilets, ATMs, handrails, playgrounds and outdoor gyms were identified as fomites with the highest rates of SARS‐CoV‐2 detection. Taken together, our data provide a real‐world picture of SARS‐CoV‐2 dispersion in highly populated tropical areas and identify critical control points that need to be targeted to break SARS‐CoV‐2 transmission chains.

## Introduction

Coronaviruses (CoVs) are members of the *Coronaviridae* family and represent a diverse group of viruses that cause respiratory and intestinal infections in animals and humans (Fehr and Perlman, 2015). The *Orthocoronavirinae* subfamily is divided into four genera – *Alphacoronavirus*, *Betacoronavirus*, *Gammacoronavirus*, and *Deltacoronavirus*. Alphacoronaviruses (HCoV‐229E and HCoV‐NL63) and betacoronaviruses (HCoV‐OC43 and HCoV‐HKU1) are commonly associated with mild respiratory disease in humans (Cui *et al*., 2019). However, in the last two decades, three highly pathogenic betacoronaviruses have emerged from animal sources to cause severe respiratory disease in humans: severe acute respiratory syndrome coronavirus (SARS‐CoV) (Zhong *et al*., 2003), Middle East respiratory syndrome coronavirus (Zaki *et al*., 2012), and more recently, the severe acute respiratory syndrome coronavirus 2 (SARS‐CoV‐2) (Lu *et al*., 2020; Zhu *et al*., 2020; Zhou *et al*., 2020b).

SARS‐CoV‐2 first emerged in the city of Wuhan, Hubei province, China, in December 2019 causing an outbreak of a yet unknown acute pneumonia (Huang *et al*., 2020). The new coronavirus was found to be highly transmissible among humans and has spread rapidly around the globe prompting the World Health Organization (WHO) to declare a pandemic on March 11, 2020 (Petersen *et al*., 2020). As of September 30, 2021, there have been approximately 233.6 million confirmed cases of COVID‐19 across the world, with over 4.7 million deaths (Dong *et al*., 2020). Difficult to control viral transmission allied with the slow progress in the rollout of COVID‐19 vaccines in most countries have contributed to the emergence of new variants of concern of SARS‐CoV‐2, which are more transmissible and can escape from natural and vaccine‐acquired immunity (Abdool Karim and De Oliveira, 2021; Faria *et al*., 2021; Naveca *et al*., 2021; Peacock *et al*., 2021; Silva and Pena, 2021).

SARS‐CoV‐2 is spread from person to person mainly through exposure to respiratory fluids containing infectious virus. Virus exposure can occur in three main ways, which are not mutually exclusive: (i) inhalation of infectious virus present in very small fine droplets and aerosol particles; (ii) deposition of virus on exposed mucous membranes in the mouth, nose or eye by direct splashes and sprays, and (iii) touching mucous membranes with hands contaminated by exhaled respiratory fluids containing virus or from touching fomites containing the virus (Marquès and Domingo, 2021; CDC, 2021b). Notably, SARS‐CoV‐2 has been found to have high person‐to‐person transmission through direct contact with infected individuals (Hu *et al*., 2021), especially by coughing, sneezing and even breathing/talking by an infected person (Tang *et al*., 2013; Kutter *et al*., 2018; Leung *et al*., 2020; Stadnytskyi *et al*., 2020). SARS‐CoV‐2 enters the body through the mucous membranes of the eyes, mouth or nose and spreads to the nose line, sinus cavity and throat until deposition into the human respiratory tract (Kaur *et al*., 2020). Although transmission through direct contact, or airborne (respiratory droplets and/or aerosols) are considered the dominant routes for the spread of SARS‐CoV‐2 (Falahi and Kenarkoohi, 2020; Zhang *et al*., 2020), some epidemiological studies have found fomites as a possible source of infection (Brlek *et al*., 2020; Luo *et al*., 2020; Xie *et al*., 2020). The risk of infection by environmental surfaces is influenced by the distance from the source, the amount of virus to which a person is exposed and the length of time since the virus has been deposited on the surface, since SARS‐CoV‐2 viability over time is influenced by environmental factors such as type of surfaces, temperature, humidity and ultraviolet radiation (e.g. sunlight) (Chin *et al*., 2020; Zhang *et al*., 2020; Van Doremalen *et al*., 2020). Thus, understanding distribution and patterns of environmental contamination by SARS‐CoV‐2 are relevant information for public health authorities. This knowledge allows the identification of critical points to establish effective control measures and may provide useful data to estimate silent circulation of SARS‐CoV‐2.

Several recent studies have investigated the presence of SARS‐CoV‐2 RNA in air and environmental surfaces, especially in health care settings (Chia *et al*., 2020; Colaneri *et al*., 2020; Liu *et al*., 2020; Mouchtouri *et al*., 2020; Santarpia *et al*., 2020; Tan *et al*., 2020; Ye *et al*., 2020; Zhou *et al*., 2020a; Dargahi *et al*., 2021). Previous studies under controlled laboratory conditions have demonstrated the ability of SARS‐CoV‐2 to remain infectious on different types of common surfaces, such as stainless steel, glass and paper, for up to 28 days at 20°C (Riddell *et al*., 2020), and it can also remain infectious in aerosols for up to 3 h (Van Doremalen *et al*., 2020). However, little is known about SARS‐CoV‐2 contamination of environmental surfaces in tropical public areas with a large flow and concentration of people. Therefore, studies investigating the presence of SARS‐CoV‐2 RNA on surfaces, and the infectious potential of these particles are of paramount importance.

To address this gap of knowledge, we investigated the presence of SARS‐CoV‐2 RNA on highly touched surfaces in Recife, a large city in Pernambuco state with a tropical monsoon climate. Samples were collected during the second wave of the COVID‐19 in Brazil, one of the most severely affected countries by the pandemic (Silva and Pena, 2021). Our findings showed widespread viral contamination across many urban public settings and poor adherence to COVID‐19 mitigation measures. Taken together, our data provide a real‐world picture of SARS‐CoV‐2 dispersion in public areas and identify critical control points that need to be targeted to halt SARS‐CoV‐2 transmission.

## Materials and methods

### Study design and setting

This cross‐sectional study was conducted in Recife, the capital of Pernambuco state, which is one of the most densely populated metropolitan regions in Brazil with 1 537 704 million people (https://cidades.ibge.gov.br/brasil/pe/recife). The city is located on the coast of Northeast coast of Brazil and has a tropical monsoon climate under the Köppen climate classification, with warm to hot temperatures and high relative humidity throughout the year. To design the study, we selected the main public places of the city that have intense circulation and concentration of people.

**Fig. 1:**
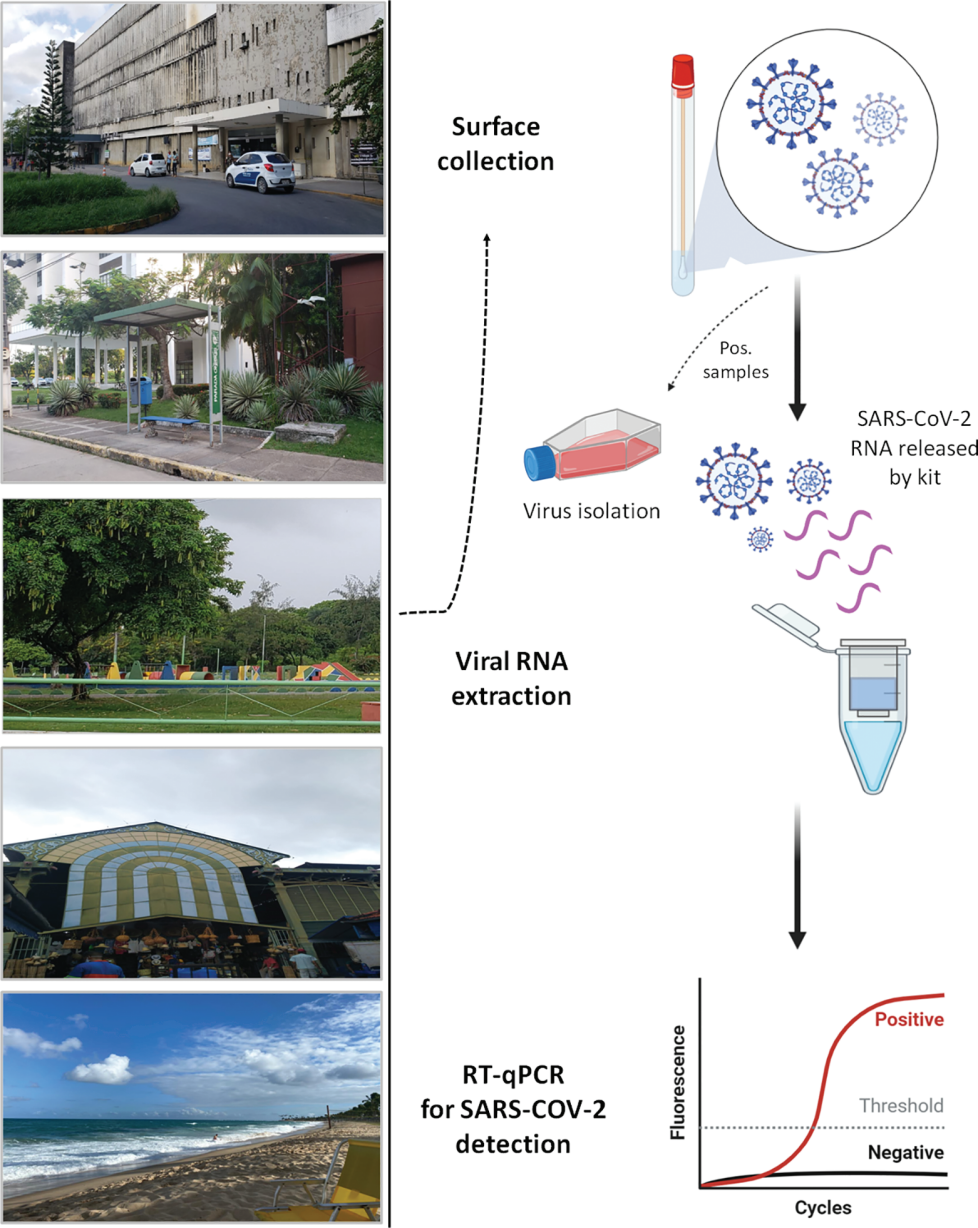
Study design showing the collection points of surface samples and the graphical workflow used to test the swabs.

**Fig. 2:**
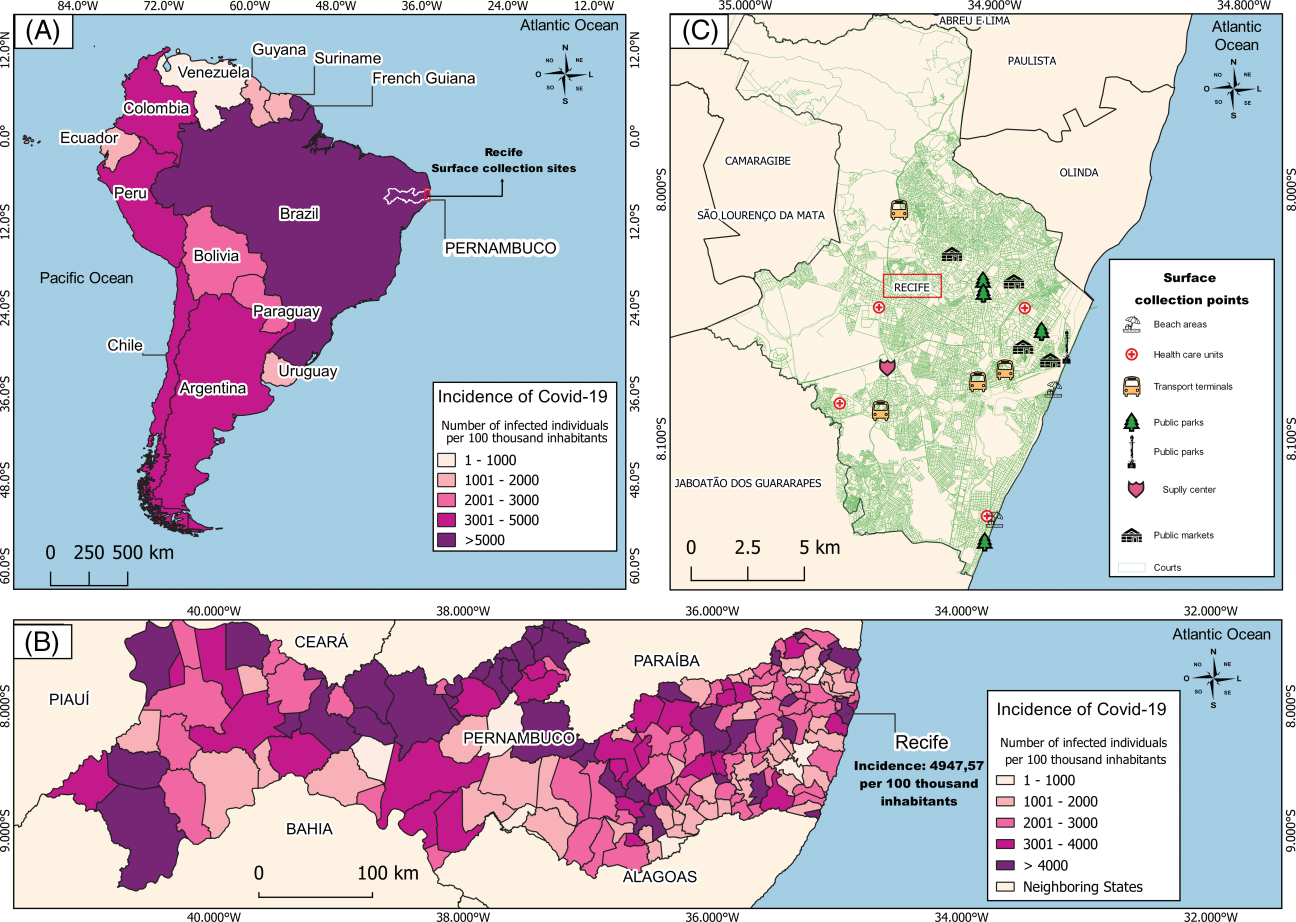
Spatial distribution of surface collection points and incidence of COVID‐19 in Latin America and Pernambuco state, Brazil. A. The incidence of COVID‐19 per 100 000 inhabitants in Latin America. B. The incidence of COVID‐19 per 100 000 inhabitants in all cities in the state of Pernambuco, Northeast Brazil. C. The spatial distribution of surface collection points (transport terminals, health care units, beach areas, public parks, supply centre and public markets) across Recife, Pernambuco state, Brazil.

Initially, we subdivided Recife's highly frequented areas into six categories, including: (i) transport terminals; (ii) health care units; (iii) beach areas; (iv) public parks; (v) supply centre; and (vi) public markets. A total of 400 environmental surface specimens were collected between February 2 and February 25, 2021 (Fig. 1). Samples were collected between 9:00 a.m. and 1:00 p.m. During sample collection, the temperature was between 26°C and 32°C (average temperature 29°C) and the average humidity was 72%. Environment data were obtained from the Time and Date AS website (http://www.timeanddate.com/weather/brazil/recife/climate). This coincided with a period of progressive increase in the number of COVID‐19 cases in Pernambuco state and Brazil, representing the ascendant phase of the second wave during the COVID‐19 pandemic course in this part of the world and also the beginning of COVID‐19 vaccination efforts in this state (Fig. 2). The ongoing pandemic of COVID‐19 in the Pernambuco state has resulted in 620 723 laboratory‐confirmed cases and 19 740 deaths as of 30 September 2021. It is important to highlight that Recife has a high concentration of specialized hospitals and is considered a reference health centre for the Northeast region of Brazil.

### Sampling areas

#### Transport terminals

A total of 84 surface samples were collected from four public transport terminals with a large daily passenger flow and concentration. We strategically selected transport terminals that connect several cities in the metropolitan region of Recife. Twenty‐one swabs were collected for each transport terminal. The collection points included the external area of the transport terminal and neighbouring areas: (i) bus terminal entrance; (ii) bus terminal exit; (iii) bus terminal access; (iv) subway station access; (v) ATM; (vi) toilet; (vii) handrail; (viii) bench; (ix) bus stop; (x) counter; (xi) faucet; (xii) ticket machine.

#### Health care units

A total of 84 surface samples were collected from four reference hospitals for the treatment of COVID‐19 patients in Recife, Brazil. Twenty‐one swabs were collected for each hospital. The collection points included the external area of the hospital and neighbouring areas: (i) principal entrance; (ii) hospital access; (iii) ambulatory entrance; (iv) patient sample collection area; (v) toilet; (vi) traffic light button; (vii) coffee shop; (viii) public phone; (ix) bus stop; (x) resting area.

#### Beach areas

A total of 21 surface samples were collected from two beaches located in the coastal area of Recife, Brazil. Interestingly, the visited beaches had a high concentration of people during the time of surface collection and during all times of restrictive relaxation measures established by the state government during the COVID‐19 pandemic. The collection points included: (i) toilets; (ii) benches; (iii) public bike station; (iv) outdoor gym; (v) fresh green coconut; (vi) handrails; (vii) faucet; (viii) traffic light button; (ix) bus stop; (x) resting area.

#### Public parks

A total of 105 surface samples were collected from five public parks. We strategically selected parks with high visitor flow, including children who access the playground. Twenty‐one swabs were collected for each public park. The collection points included: (i) playground; (ii) recreation area; (iii) outdoor gym; (iv) toilet; (v) handrail; (vi) bus stop; (vii) public bike station; (viii) traffic light button; (ix) coffee shop; (x) faucet.

#### Supply centre

A total of 21 surface samples were collected from one food distribution centre located in Recife, Brazil. We selected this place as it is a place which serves as a gateway for people from all over the Brazilian territory, and acts as a source of food supply for the Northeast of Brazil. The collection points included: (i) toilet; (ii) restaurant; (iii) handrail; (iv) resting area.

#### Public markets

A total of 85 surface samples were collected from four public markets. Twenty‐one swabs were collected for each public market with exception of one, where we collected 22 swabs. The collection points included: (i) principal entrance; (ii) side entrance; (iii) public market access; (iv) toilet; (v) kiosk; (vi) store; (vii) food hall; (viii) traffic light button; (ix) faucet; (x) resting area; (xi) outside area.

### Surface sampling

Environmental samples were collected by qualified technicians who had received biosafety training and were equipped with personal protective equipment. For sample collection, sterile swabs (bioBoa Vista, Brazil) were used, which were put into a conical tube (15 ml) containing 2 ml of virus preservation solution (sterile phosphate‐buffered saline, pH 7.2). Each swab was vigorously rubbed on the surface with a collection area of 25 cm^2^. Samples were collected from distinct types of materials, including metal, plastic, wood, rock, concrete, and glass. The time of collection and climate conditions of the day were recorded during sampling. In addition, an environmental site assessment questionnaire was filled by technicians at each sample collection point with the aim to identify whether the collection environment and the population were following public health measures for preventing the rapid spread of SARS‐CoV‐2 and, subsequently, the COVID‐19 transmission.

### Sample transfer and processing

Surface samples were collected and immediately stored at 4°C prior to transfer to the Biosafety Level 3 Laboratory (BSL‐3) of Fiocruz Pernambuco, Brazil, where all samples were processed until 72 h after collection with aim to preserve the infectivity or genome integrity of SARS‐CoV‐2. After processing, each sample was taken directly tested according to the instructions described below.

### Viral RNA extraction and RT‐qPCR for SARS‐CoV‐2 detection

Viral RNA was extracted from surface samples (140 μl of transport solution) using the QIAamp Viral RNA Mini Kit (QIAGEN, Germany) following the manufacturer's protocol. RT‐qPCR assay targeting the N protein according to protocols recommended by the Centres for Disease Control and Prevention – CDC USA was used to detect SARS‐CoV‐2 (Supplementary Table 1) (CDC, 2020). Samples were considered positive when they presented amplification for N1 target, considering the threshold for cycle quantification (Cq) value of 40 (CDC, 2020). Samples with Cq ≥40 were considered negative. Briefly, each reaction was prepared using the QuantiNova Probe RT‐PCR Kit (QIAGEN, Valencia, CA, USA) following the manufacturer's protocol and the CDC‐USA recommendations in a total volume of 10 μl. Negative (extraction control and non‐template control) and positive controls (RNA extracted from SARS‐CoV‐2 cell supernatants) were included during all experiments. Primer and probe sequences were synthetized by IDT (Integrated DNA Technologies, Skokie, IL, USA). Thermal cycling was performed at 45°C for 15 min for reverse transcription, followed by 95°C for 5 min and then 45 cycles of 95°C for 03 s and 55°C for 30 s. All experiments were conducted using the Applied Biosystems QuantStudio 5 Real‐Time PCR Systems (Applied Biosystems, USA). For data analysis, the QuantStudio software v1.5 was used with baseline and threshold automatic.

### Cells

African monkey green kidney‐derived cell line Vero CCL‐81 was used for virus isolation from positive environmental samples. Cells were cultured in Dulbecco's modified Eagle's medium, high glucose (Gibco, USA) supplemented with 10% heat‐inactivated foetal bovine serum, 100 U ml^−1^ penicillin and 100 μg ml^−1^ streptomycin (Gibco); and maintained in a humidified atmosphere, at 37°C and 5% CO_2_.

### SARS‐CoV‐2 isolation

Vero CCL‐81 cells were cultured in 12‐well plates at a density of 2 × 10^5^ cells/well. After 24 h, the culture media was removed and cells were incubated with 300 μl of undiluted and filtered surface samples at 37°C, 5% CO_2_, for 1 h. Fresh media supplemented with 2% FBS (700 μl) was added to the cells and they were maintained at 37°C, 5% CO^2^. Cells were monitored daily for the visualization of virus‐induced cytopathic effect (CPE). CPE images were acquired in Carl Zeiss Axio Observer 5 microscope coupled to a photographic camera. After 3 days post‐infection supernatants were collected and 300 μl were transferred to a new 12‐well plate. This procedure was repeated until completing three passages (P1, P2 and P3). Following this, cell culture supernatants were collected on *t* = 0 h and *t* = 72 h in each passage for viral RNA extraction and possible SARS‐CoV‐2 detection by RT‐qPCR. All experiments were performed in a BSL‐3 facility.

### Environmental site assessment questionnaire

Data regarding the social distancing, mask wearing, availability of hand sanitizers and COVID‐19 control measures during sample collection in all locations were obtained using a structured questionnaire following the recommendations and guidelines established by WHO and CDC (CDC, 2021a). The questions aimed to identify the implementation and compliance with COVID‐19 prevention measures, including social distancing, mask wearing, the availability of hand sanitizers, body temperature measurements for screening and the presence of informative charts for people education. The questionnaires were made with qualitative, with ‘yes’ or ‘no’ input, or quantitative inquiries.

### Spatial location of collection surfaces

To georeference the locations where surface samples were obtained, we used the QGIS software (https://qgis.org/en/site/) to generate a map using the geographic coordinates of each publicly available location at https://www.google.com.br/maps. First, we created a graduate map with information about the incidence of COVID‐19 in the countries of Latin America (Fig. 2A) and all cities located in the State of Pernambuco, Brazil (Fig. 2B). The incidence per 100 thousand inhabitants was calculated using the database of the last Brazilian census available at http://censo2010.ibge.gov.br and epidemiological reports of COVID‐19 cases from the Pernambuco State Health Department (SAÚDE, 2021) and the World Organization Health (WHO) (ORGANIZATION, 2021). Furthermore, we showed the spatial distribution of urban public places where the samples were collected including transport terminals, health care units, public parks, public markets, beach areas and food supply centre. We acquired the cartographic base in shapefile format through the Brazilian Institute of Geography and Statistics (IBGE) in the Geocentric Reference System for the Americas (SIRGAS) 2000 (Fig. 2C).

### Data analysis

GraphPad Prism software version 5.01 for Windows (GraphPad Software, La Jolla, CA, USA) was used to plot most graphics. The association analysis between collection locations and type of materials was demonstrated based on the results from 97 positive surfaces collected in this study using the web‐based Circos table viewer, version 0.63‐9 (https:www.mkweb.bcgsc.ca/tableviewer) (Krzywinski *et al*., 2009).

### Ethics approval

This study was reviewed and approved under protocol number 03/2021 by the Fiocruz Pernambuco Internal Biosafety Commission, as part of quality assurance for working with highly pathogenic virus.

## Results

### Distribution of surface samples according to collection area and type of material

**Fig. 3:**
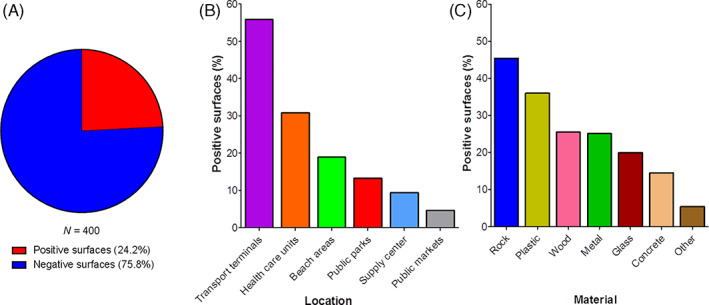
Overall results for SARS‐CoV‐2 detection in surface samples. A. The distribution of positive and negative samples using a total of 400 environmental samples. B. The distribution of positive samples according to the collection areas, including transport terminals, health care units, beach areas, public parks, supply centre and public markets. C. The distribution of positive samples according to the type of material including metal, plastic, wood, rock, concrete, glass and others.

A total of 400 surface samples were collected in Recife, Pernambuco state in 19 sites divided into six subgroups (health care units, transport terminals, public parks, public markets, beach areas and a food supply centre). A total of 97 surface samples (24.2%) tested positive for SARS‐CoV‐2 RNA using the CDC‐USA protocol by RT‐qPCR (Fig. 3A, Supplementary Fig. 1) in 18 out (94.7%) of 19 sites sampled (Table 1). The only site that tested negative was a public market. SARS‐CoV‐2 RNA was detected mainly around transport terminals (47/84, 55.9%), followed by health care units (26/84, 30.9%), beach areas (4/21, 19.0%), public parks (14/105, 13.3%), food supply centre (2/21, 9.5%) and public markets (4/85, 4.7%). (Fig. 3B, Supplementary Table 2). Regarding the type of material where environmental samples were collected, SARS‐CoV‐2 RNA was found most frequently on rock (10/22, 45.4%), followed by plastic (18/50, 36.0%), wood (12/47, 25.5%), metal (45/179, 25.1%), glass (2/10, 20.0%), concrete (8/55, 14.5%) and other (ceramic and rubber) (2/37, 5.4%) (Fig. 3C, Supplementary Table 2). Positive samples were predominantly found in toilets, ATMs, handrails, playground and outdoor gym; highlighting the importance of these fomites in SARS‐CoV‐2 surface contamination.

**Table 1:** Positivity of SARS‐CoV‐2 RNA on public touched surfaces at different locations in Recife, Pernambuco state, Brazil.

Location	Site #	Number of samples collected	Number of positive samples (%)
Transport terminals	01	21	20 (95.2%)
	02	21	16 (76.1%)
	03	21	8 (38.0%)
	04	21	3 (14.2%)
Health care units	05	21	4 (19.0%)
	06	21	3 (14.2%)
	07	21	18 (85.7%)
	08	21	1 (4.76%)
Public parks	09	21	3 (14.2%)
	10	21	4 (19.0%)
	11	21	3 (14.2%)
	12	21	2 (9.52%)
	13	21	2 (9.52%)
Public markets	14	21	2 (9.52%)
	15	21	0 (0%)
	16	21	1 (4.76%)
	17	22	1 (4.54%)
Beach areas	18	21	4 (19.0%)
Supply centre	19	21	2 (9.52%)

### Distribution of positive surface samples according to point of collection

#### Transport terminals

**Fig. 4:**
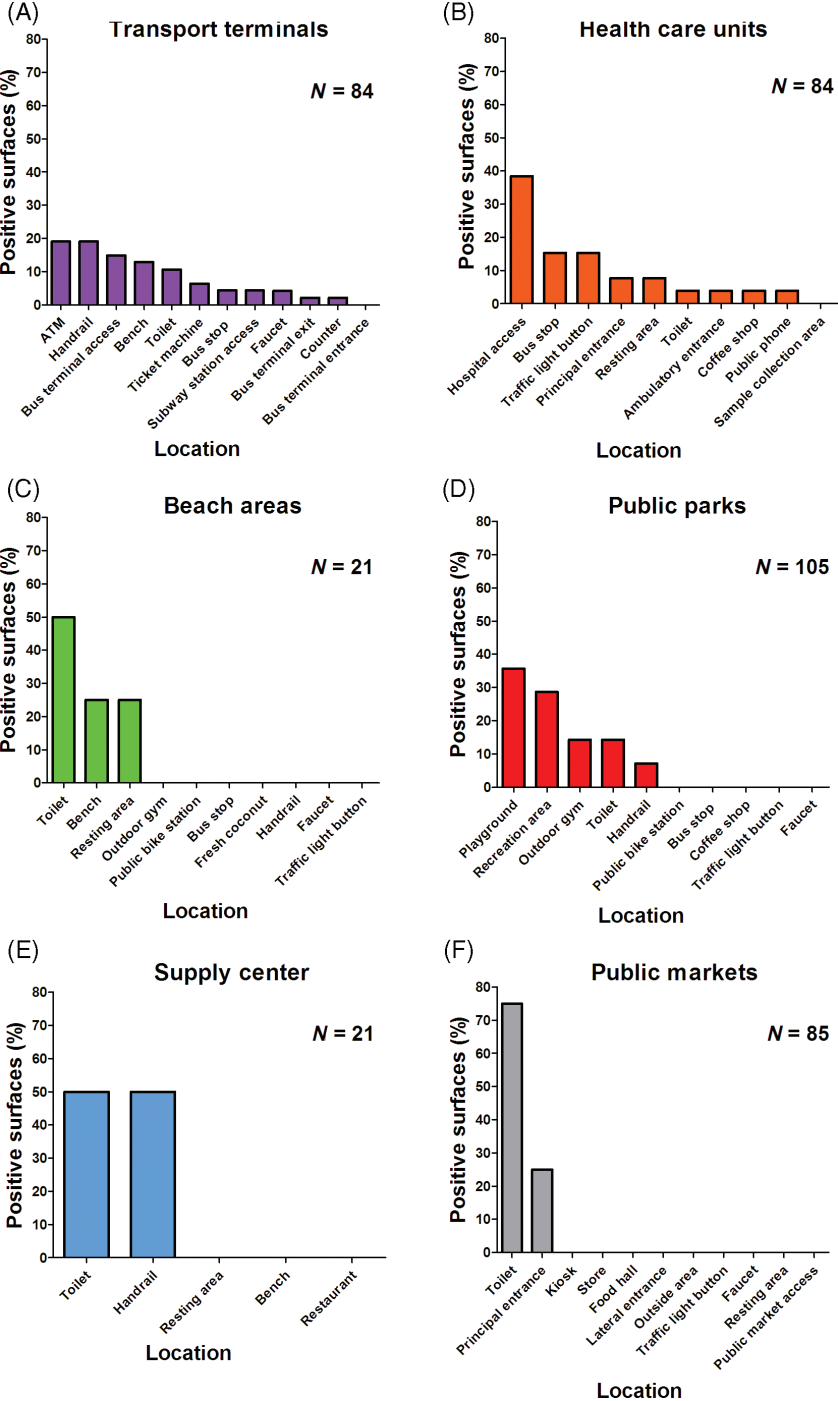
Distribution of positive surface samples according to collection areas. A. The distribution of positive samples in transport terminals. B. The distribution of positive samples around health care units. C. The distribution of positive samples in beach areas. D. The distribution of positive samples in public markets. E. The distribution of positive samples in a supply centre. F. The distribution of positive samples in public markets.

Forty‐seven (48.4%) surface samples were positive for SARS‐CoV‐2 RNA around public transport terminals with Cq values ranging from 31.1 to 38.7 by RT‐qPCR (Supplementary Table 2). Positive samples were distributed particularly in 11 different locations, including ATM (9/47, 19.1%), handrails (9/47, 19.1%), bus terminal access (7/47, 14.8%), bench (6/47, 12.7%), toilet (5/47, 10.6%), ticket machine (3/47, 6.3%), bus stop (2/47, 4.2%), subway station access (2/47, 4.2%), faucet (2/47, 4.2%), bus terminal exit (1/47, 2.1%) and ticket counter (1/47, 2.1%) (Fig. 4A, Supplementary Table 2).

#### Health care units

Twenty‐six (26.8%) surface samples were positive for SARS‐CoV‐2 RNA in the surroundings of health care units with Cq values ranging from 31.1 to 38.7 by RT‐qPCR (Supplementary Table 2). Positive samples were found in nine different locations from four reference hospitals for COVID‐19 treatment. The areas with highest number of positive samples were hospital access (10/26, 38.4%), bus stop (4/26, 15.3%), traffic light button (4/26, 15.3%), principal entrance (2/26, 7.6%), resting area (2/26, 7.6%), toilet (1/26, 3.8%), ambulatory entrance (1/26, 3.8%), coffee shop (1/26, 3.8%) and public phone (1/26, 3.8%) (Fig. 4B, Supplementary Table 2).

#### Beach areas

Four (4.1%) surface samples were positive for SARS‐CoV‐2 RNA in beach areas with Cq values ranging from 36.1 to 37.9 by RT‐qPCR (Supplementary Table 2). Positive samples were collected from three different locations, including toilets (2/4, 50.0%), bench (1/4, 25.0%) and resting area (1/4, 25.0%) (Fig. 4C, Supplementary Table 2). No positive samples were detected from the outdoor gym, public bike station, bus stop, fresh coconut, handrail, faucet, or traffic light button.

#### Public parks

Fourteen (14.4%) surface samples were positive for SARS‐CoV‐2 RNA around public parks, with Cq values ranging from 36.2 to 39.7 by RT‐qPCR (Supplementary Table 2). Positive samples were collected from five different locations, including playground (5/14, 35.7%), recreation area (4/14, 28.5%), outdoor gym (2/14, 14.2%), toilet (2/14, 14.2%) and handrails (1/14, 7.1%) (Fig. 4D, Supplementary Table 2). There were no positive samples from the public bike station, bus stop, coffee shop, traffic light button, or faucet.

#### Supply centre

Two (2.0%) surface samples were positive for SARS‐CoV‐2 RNA in a food distribution centre with Cq values ranging from 38.0 to 38.7 by RT‐qPCR (Supplementary Table 2). Positive samples were collected from two different locations, including toilet (1/2, 50.0%) and handrails (1/2, 50.0%) (Fig. 4E, Supplementary Table 2). No positive samples were found in restaurants or resting benches.

#### Public markets

Three out of four public markets sampled returned at least one positive sample. Four (4.1%) surface samples were positive for SARS‐CoV‐2 RNA in public markets with Cq values ranging from 36.9 to 38.1 by RT‐qPCR (Supplementary Table 2). Positive samples were collected from two different locations, including toilets (3/4, 75.0%) and principal entrance (1/4, 25.0%) (Fig. 4F, Supplementary Table 2). No positive samples were found at the kiosk, store, lateral entrance, outside area, food hall, public market access, traffic light button, faucet, or resting area.

### Types of surface materials positive for SARS‐CoV‐2 RNA

**Fig. 5:**
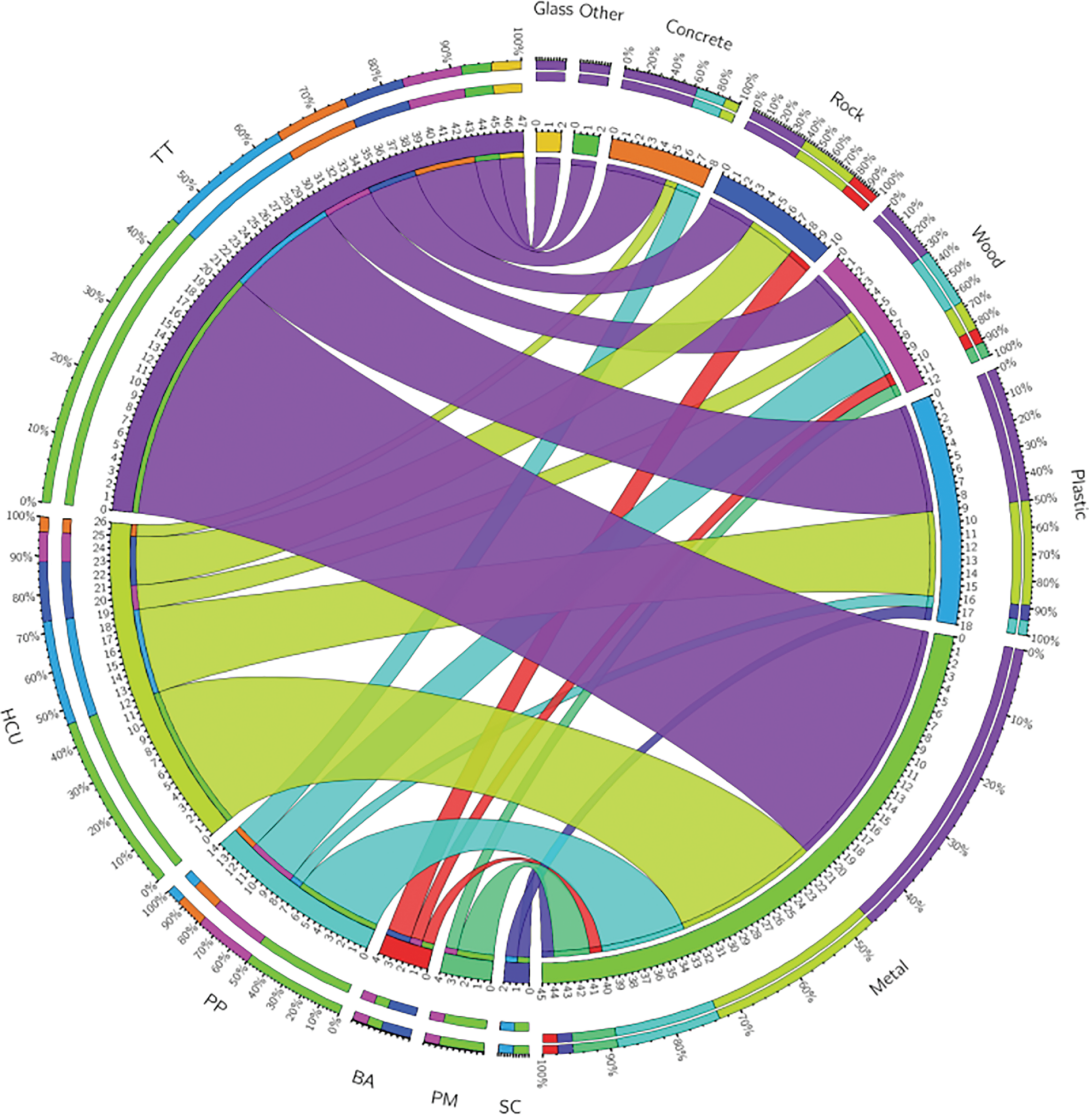
Association between the surface collection areas and type of material where SARS‐CoV‐2 RNA was detected. TT: transport terminals; HCU: health care units; PP: public parks; BA: beach areas; PM: public markets; SC: supply centre.

From the 47 positive samples in transport terminals, 21 (44.6%) samples were identified mainly on metal surfaces, especially from handrails at bus terminals, ATM buttons, protection grids, and faucets. Nineteen (19.1%) samples were recovered from plastic surfaces, especially around biometrics sensors in ATMs and faucets in the toilet. Five (10.6%) samples were found in concrete surfaces, most being found in pillars near the bus stop and one sampled from a bench. Four (8.5%) samples were collected on rock surfaces, with virus being detected on walls in the toilet and bus terminal, and one sample was collected at the terminal service desk. Four (8.1%) samples were identified on wood surfaces, all being from benches near the bus stop of transport terminals. Two (4.2%) samples were detected on glass surfaces, mainly on the ticket machine screens. In addition, one (2.1%) sample was collected on a toilet seat (porcelain) and one (2.1%) was detected on the ticket machine (rubber) (Fig. 5, Supplementary Table 2).

From the 26 positive samples found in health care units and neighbouring areas, 12 (46.1%) samples were recovered from metal surfaces mostly, located at the entrance to hospitals and near bus stops. Seven (26.9%) samples were identified in plastic surfaces, especially from traffic light buttons, near bus stops and in the toilets. Four (15.3%) samples were detected in rock surfaces found at the entrance to hospitals. Two (7.6%) samples were identified in wood surfaces at the entrance to hospitals. One (3.8%) sample was detected on the concrete surface from a nearby bus stop (Fig. 5, Supplementary Table 2).

From the 14 positive samples found in public parks, seven (50.0%) samples were identified on the metal surfaces of handrails in the playground and outdoor gym. Four (28.5%) samples were recovered from wood surfaces in the playground, and one tourist attraction point. Two (14.2%) samples were detected in concrete surfaces of the playground. One (7.1%) sample was identified in the plastic surface from a faucet in the toilet (Fig. 5, Supplementary Table 2).

From the four positive samples in beach areas, two (50.0%) were detected in rock surfaces, one from toilet wall and one from a bench. One (25.0%) sample was identified in a metal surface from a faucet in the toilet, and a further one (25.0%) was detected in a wood surface on a handrail that gives access to the beach (Fig. 5, Supplementary Table 2).

From the four positive samples in public markets, three (75.0%) samples were detected on metal surfaces at the entrance to public markets, and from a toilet faucet. One (25.0%) sample was identified in wood surfaces from a door in the toilet (Fig. 5, Supplementary Table 2).

Lastly, from the two positive samples from one food distribution centre, one (50.0%) sample was detected on a plastic surface from a faucet in the toilet and one (50.0%) was identified on a metal handrail surface at the entrance of a bank (Fig. 5, Supplementary Table 2).

### Viability of SARS‐CoV‐2 from positive surfaces samples

To assess infectivity of samples that tested positive by RT‐qPCR, nine samples with Cq value <34 (Cq ranging from 31.0 to 33.7) were inoculated into 12‐well plates seeded with Vero CCL‐81 cells. Samples were considered negative after three blind passages of the supernatant. Under these conditions it was not possible to isolate the virus, as determined by the absence of CPE and negative RT‐qPCR results from the third passage supernatant (Supplementary Fig. 2, Supplementary Table 3). The risk of infection from these contaminated surfaces is therefore not clear.

### Poor adherence of COVID‐19 mitigation measures by society

Data regarding the adoption of public health measures and community perception of COVID‐19 disease were collected during surface collection in all locations by using a structured environmental site assessment questionnaire. In the 19 collection points, 70% alcohol‐based hand sanitizer was available at the entrance in 26.3% (5/19) of the locations, whereas 42.1% (8/19) had a sink with soap and water for hand hygiene. Temperature measurements at the entrance were carried out in 15.8% (3/19) of the sites, and information material on preventive measures to prevent SARS‐CoV‐2 transmission was found in 42.1% (8/19) of the sites. High mask wear adherence was seen [94.7% (18/19)], although only 57.3% of people (average calculated for every 10 people per collection point) were wearing masks in a proper way. Regarding social distancing, only 26.3% (5/19) of the people present at collection points were maintaining the recommended social distance of 2 m. Furthermore, only 5.3% (1/19) of collection sites were limiting the number of people who accessed the location point (Supplementary Table S4). We found no positive correlation between adherence of COVID‐19 mitigation measures and SARS‐CoV‐2 positivity (data not shown). Overall, our findings indicated poor adherence of COVID‐19 mitigation measures in our study areas.

## Discussion

Since the emergence of SARS‐CoV‐2, first identified in China, the highly pathogenic coronavirus has spread rapidly around the world causing an unprecedented health security crisis and drastically affecting the global economic stability. Thus, understanding the modes of transmission of SARS‐CoV‐2 among humans is a critical step to establish effective prevention policies and prioritize resources to break the chain of SARS‐CoV‐2 transmission. The transmission through direct contact and via airborne (respiratory droplets and/or aerosols) are pointed as the dominant routes for the transmission of SARS‐CoV‐2 in humans (Falahi and Kenarkoohi, 2020; Zhang *et al*., 2020; Greenhalgh *et al*., 2021) and animal models, like ferrets (Richard *et al*., 2020), golden hamsters (Sia *et al*., 2020), and mice (Bao *et al*., 2020). However, some epidemiological reports have found fomites as a possible source of infection (Brlek *et al*., 2020; Luo *et al*., 2020; Xie *et al*., 2020). The transmission dynamics of SARS‐CoV‐2 by environmental surfaces and their role in the transmission chain remain unclear and may be multifactorial, especially in urban areas with a large flow of people with real‐life challenges. Here, we investigated the presence of SARS‐CoV‐2 RNA on public high‐touch surfaces in a large metropolitan city during a relevant section of the second wave of the COVID‐19 pandemic course in Brazil. Our findings represent specific collection points of the city and its specific conditions at the time of sampling.

A recent study investigated the presence of SARS‐CoV‐2 RNA on public surfaces in Belo Horizonte, a large city with a tropical savanna climate in Southeast Brazil using a total of 933 surface specimens from different locations (health care units, public squares, bus terminals, public markets and other public places) were collected between April and June 2020 (Abrahão *et al*., 2021). The results showed that 49 (5.25%) of surface samples were tested positive for SARS‐CoV‐2 RNA, although the infectious potential of positive samples was not investigated. Considering the proportion of positivity in the different places, the authors pointed out that bus terminals exhibited the highest positivity rate, followed by public markets, public squares and health care units (Abrahão *et al*., 2021). In our study, we found higher positivity of SARS‐CoV‐2 RNA (97/400, 24.2%) when compared to the Belo Horizonte survey. Moreover, most of the positive samples in our study were detected in the surroundings of public transport terminals (47/84, 55.9%), followed by health care units (26/84, 30.9%), beach areas (4/21, 19.0%), public parks (14/105, 13.3%), other places (2/21, 9.5%) and public markets (4/85, 4.7%). The difference in the positivity rate of both cities cannot be explained by climate differences as Recife is hotter and more humid than Belo Horizonte, conditions that decrease the stability of SARS‐CoV‐2 in the environment (Biryukov *et al*., 2020) and its transmissibility (Qi *et al*., 2020). A more plausible explanation for this disparity is the number of confirmed COVID‐19 cases in these cities by the time of sample collection. Although Belo Horizonte reported 400–5000 (https://ciis.fmrp.usp.br/covid19/bh‐mg/) daily cases between April and June 2020, Recife had 60 000–70 000 (https://ciis.fmrp.usp.br/covid19/recife‐pe/) in February 2021.

Regarding the distribution of positive samples according to the type of material, we found the SARS‐CoV‐2 RNA mainly on rock, followed by plastic, wood, metal, glass and concrete. Previous studies performed under controlled laboratory conditions have shown that SARS‐CoV‐2 remains infectious on different types of surfaces, such as stainless steel, glass and paper, for up to 28 days at 20°C (Riddell *et al*., 2020), depending on the type of environmental surface; and can remain viable in aerosols for up to 3 h (Van Doremalen *et al*., 2020). Notably, the viral load decreases over time and depends on the length of time since the virus has been deposited on the surface, which may be reflected in the presence of infectious or non‐infectious viral particles and, consequently, infection risk in humans in field settings (Chin *et al*., 2020; Zhang *et al*., 2020; Van Doremalen *et al*., 2020). Other studies have suggested that several environmental stressors can compromise and damage the integrity of SARS‐CoV‐2 viral particles, including temperature and relative humidity (Biryukov *et al*., 2020; Riddell *et al*., 2020). Additionally, our data demonstrated that the positive samples for SARS‐CoV‐2 RNA were mainly collected in toilets. This finding also corroborates with outcomes obtained by other research groups (Chia *et al*., 2020; Liu *et al*., 2020; Luo *et al*., 2020), which pointed toilets as an area of high positivity rate for SARS‐CoV‐2 RNA. Taken together, our findings revealed other specific locations with high rates of positivity: ATMs, handrails, playgrounds and outdoor gyms.

In order to elucidate the transmission dynamics of SARS‐CoV‐2 by environmental surfaces in real‐life conditions, several studies have investigated the presence of SARS‐CoV‐2 in air and environmental surfaces/areas, including health care settings (Chia *et al*., 2020; Colaneri *et al*., 2020; Liu *et al*., 2020; Mouchtouri *et al*., 2020; Santarpia *et al*., 2020; Tan *et al*., 2020; Zhou *et al*., 2020a; Dargahi *et al*., 2021) and urban settings (Cai *et al*., 2020; Di Carlo *et al*., 2020; Harvey *et al*., 2020; Luo *et al*., 2020; Abrahão *et al*., 2021). In general, these studies have found varying levels of environmental contamination, ranging from extensive (Chia *et al*., 2020; Zhou *et al*., 2020a) to low contamination (Colaneri *et al*., 2020; Abrahão *et al*., 2021), or even no contamination of SARS‐CoV‐2 RNA. However, many of these studies did not determine the ability of SARS‐CoV‐2 to be cultured from such environmental swabs, which would help to understand the implications of SARS‐CoV‐2 RNA positive environmental samples in terms of infectious potential for the human population (Santarpia *et al*., 2020; Zhou *et al*., 2020a; Rocha *et al*., 2021). Another important factor that must be considered is the minimal infectious dose of SARS‐CoV‐2 to start an effective infection in humans. A recent study estimated to be 100 viral particles, although further studies are required to confirm this infective dose (Karimzadeh *et al*., 2021).

In the current analysis, we evaluated the infectious potential of positive surface samples (Cq value <34) in Vero CCL‐81 cells, but SARS‐CoV‐2 could not be cultured. This finding is supported by recent studies, which have demonstrated the low isolation rate of infectious virus in environmental swabs (Colaneri *et al*., 2020; Mondelli *et al*., 2020; Zhou *et al*., 2020a). This may explain the lack of success in virus isolation given the short half‐life of SARS‐CoV‐2 in the environment. Serial sampling of highly touched surfaces in places with large people flow might produce culturable SARS‐CoV‐2. Nevertheless, our findings identify the locations and objects that pose the highest risk of contamination through fomites and should be considered as COVID‐19 critical control points. The difficulty in culturing viruses from environmental samples arises from low viral load concentrations and instability of SARS‐CoV‐2 outside the human host. Recent studies aggregated environmental sampling has shown high RT‐qPCR Cq values (>30) for most of the positive samples, which may explain the difficulty of SARS‐CoV‐2 to be cultured from the environmental specimens (Zhou *et al*., 2020a; Abrahão *et al*., 2021; Dargahi *et al*., 2021).

SARS‐CoV‐2 contamination of public surfaces suggests the circulation of infected people and the risk of infection in these locations either by direct or indirect contact with infected patients. Direct contact with an infectious source is important for the establishment of COVID‐19 clinical features and this has been established using animal models. Transmission studies in the ferret SARS‐CoV‐2 model have demonstrated that airborne transmission is likely but is considerably less efficient than direct contact transmission, whereby direct contacting animals are exposed to infected ferrets and share with them the same food, water, bedding and breathe the same air (Kim *et al*., 2020; Richard *et al*., 2020).

Regarding the adherence of COVID‐19 mitigation measures by society, a number of studies have been performed in order to evaluate the adoption of measures to prevent the SARS‐CoV‐2 transmission (Azene *et al*., 2020; Azlan *et al*., 2020; Shewasinad Yehualashet *et al*., 2021). To assess the community's adherence to mitigation measures to combat the rapid spread of SARS‐CoV‐2, a recent cross‐sectional study conducted in Malaysia employed 4850 Malaysian residents, between 27th March and 3rd April 2020 (Azlan *et al*., 2020). The findings revealed that most participants (83.1%) held positive attitudes toward the successful control of COVID‐19 (Azlan *et al*., 2020). Interestingly, the number of COVID‐19 cases in Malaysia remained stable, with a progressive increase observed only between September and November 2020 (https://ourworldindata.org/covid‐cases). Here, although the number of places evaluated was limited (19) and the method of answering the questionnaires was different, it is important to highlight that are places with high circulation and concentration of individuals. Our data demonstrated low adherence of COVID‐19 mitigation measures by society regarding the social distancing, precaution measures adoption and community's perception about the COVID‐19 disease. Therefore, these results highlight the importance of consistent messaging from government and health authorities to improve levels the adoption of measures to prevent and contain the spread of SARS‐CoV‐2.

In summary, our data demonstrated the extensive viral RNA contamination of surfaces in a range of public urban settings in the absence of viral isolation, which suggests low potential risk from environmental contamination for the human population. However, we identified poor adherence to COVID‐19 mitigation policies by wider society regarding the adoption of control measures, and this may be reflected in the frequent detection of the viral RNA. Studies such as these can contribute to assess the prevalence of SARS‐CoV‐2 in specific settings. Finally, we suggest that further studies are urgently performed to elucidate the relative contribution of various modes of transmission for SARS‐CoV‐2 in both healthcare and urban settings.

## Funding

The work in Dr. Pena's lab is funded by the Fiocruz Inova Program, IDRC‐Canada and the Foundation for Science and Technology of Pernambuco – FACEPE, Brazil (grant number APQ‐0560‐2.12/19). S.J.R.d.S. is supported by a PhD fellowship sponsored by the Foundation for Science and Technology of Pernambuco (FACEPE), reference number IBPG‐1321‐2.12/18. A.K. is funded by the UK Medical Research Council (MC_UU_12014/8). The funders had no role in study design, data collection and analysis, decision to publish, or preparation of the manuscript.

## Author Contribution Statement

L.P., A.K. and S.J.R.d.S. conceived the work. S.J.R.d.S., J.C.F.d.N., W.P.M.d.S.R., C.T.A.d.S., P.G.d.S., R.P.G.M., A.A.M., B.N.R.S. and J.J.F.d.M. performed the experiments. S.J.R.d.S., J.C.F.d.N., W.P.M.d.S.R., P.G.d.S. A.A.M., J.J.F.d.M., A.K. and L.P. performed data analysis and interpretation. S.J.R.d.S., J.C.F.d.N., W.P.M.d.S.R., A.A.M. and J.J.F.d.M. wrote the original draft. S.J.R.d.S., A.K. and L.P. wrote the final manuscript. L.P. supervised the work. All authors critically revised the manuscript and approved the final version of the submitted manuscript.

## Supporting information


**Appendix S1:** Supplementary Information
