# Supplementary material for: Widespread contamination of SARS‐CoV‐2 on highly touched surfaces in Brazil during the second wave of the COVID‐19 pandemic
Source: Environ Microbiol. 2022 Jan 20;23(12):7382–95. doi: 10.1111/1462-2920.15855 (PMC9303906; doi:10.1111/1462-2920.15855)
Supplement: Supplementary file 1 — Appendix S1: Supplementary Information [file EMI-23-7382-s001.docx]

**Supplementary material**

**Widespread Contamination of SARS-CoV-2 on Highly Touched Surfaces in in Brazil During the Second Wave of the COVID-19 Pandemic**

Severino Jefferson Ribeiro da Silva^1^, Jéssica Catarine Frutuoso do Nascimento^1^, Wendell Palôma Maria dos Santos Reis^1^, Caroline Targino Alves da Silva^1^, Poliana Gomes da Silva^1^, Renata Pessôa Germano Mendes^1^, Allyson Andrade Mendonça^1^, Bárbara Nazly Rodrigues Santos^1^, Jurandy Júnior Ferraz de Magalhães^1,2,3^, Alain Kohl^4^ and Lindomar Pena^1*^

^1^Laboratory of Virology and Experimental Therapy (LAVITE), Department of Virology, Aggeu Magalhães Institute (IAM), Oswaldo Cruz Foundation (Fiocruz), 50670-420, Recife, Pernambuco, Brazil.

^2^Department of Virology, Pernambuco State Central Laboratory (LACEN/PE), Recife, Pernambuco, Brazil.

^3^University of Pernambuco (UPE), Serra Talhada Campus, Serra Talhada, Pernambuco, Brazil.

^4^MRC-University of Glasgow Centre for Virus Research, Glasgow, G61 1QH, UK.

*Corresponding author: [lindomar.pena@fiocruz.br](mailto:lindomar.pena@fiocruz.br)

**Figure S1.** Standard curve using N1 and N2 primers to detect SARS-CoV-2 RNA extracted from cell supernatants.


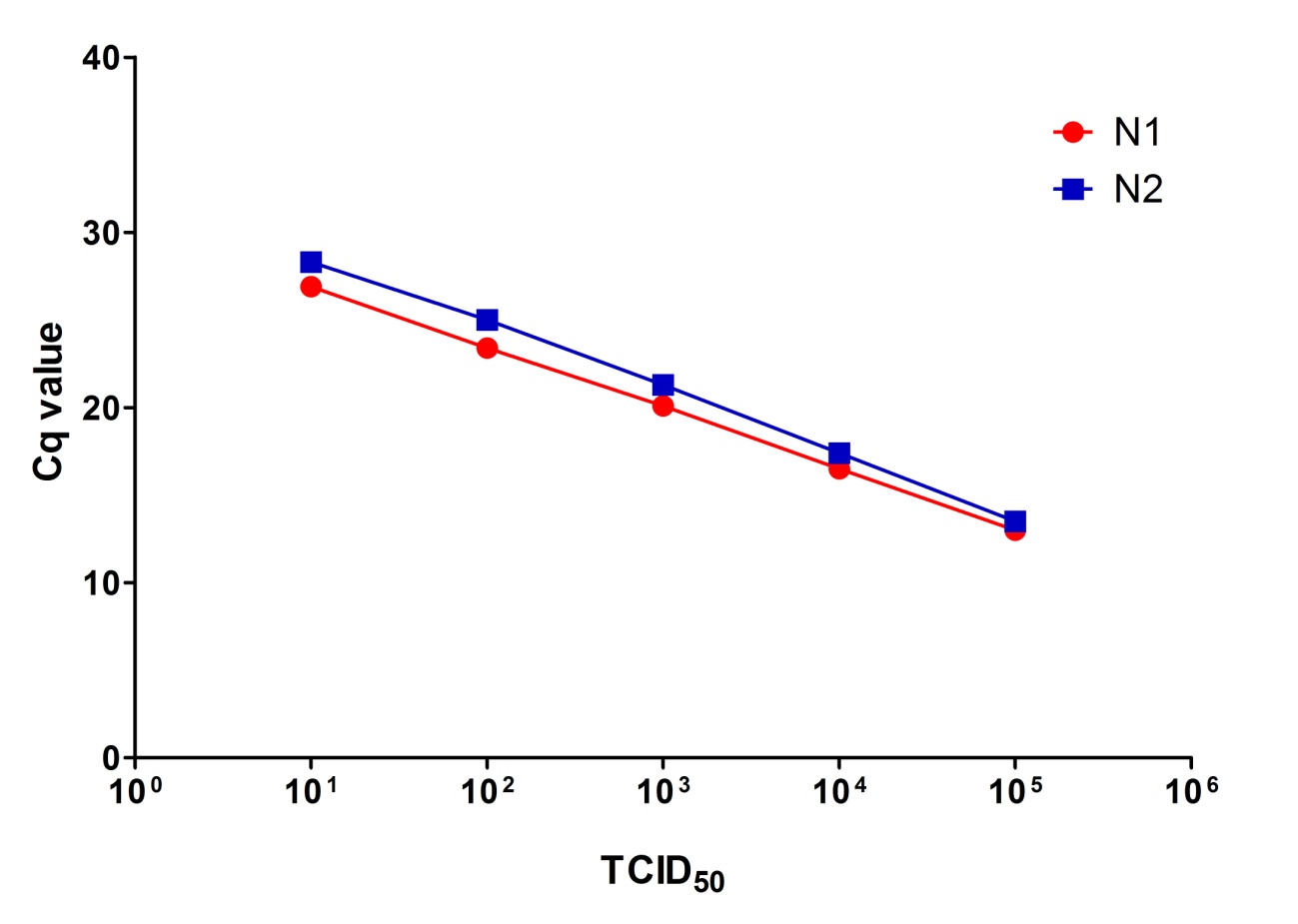


TCID_50_: 50% Tissue Culture Infectious Dose

Cq: Cycle quantification

**Figure S2.** Attempted to SARS-CoV-2 isolation using the Vero CCL-81 cell lineage.


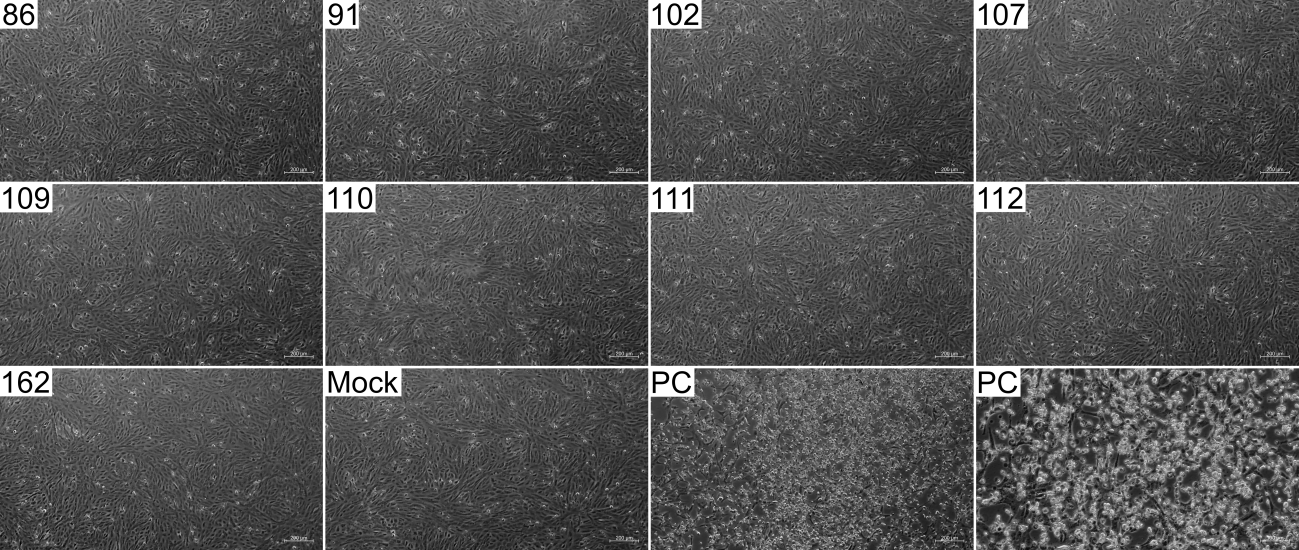


**Cytopathic effect observed in positive surface samples.** Specimens with a lower Cq value (86, 91, 102, 107, 109, 110, 111, 112 and 162) among samples tested positive for SARS-CoV-2 after three passages. Mock: uninfected cells; PC: positive control (SARS-CoV-2 isolated from patient sample).

**Table S1.** Primers used in this study for SARS-CoV-2 detection.

| **Target** | **Primer name** | **Oligonucleotide Sequence (5’-3’)^a^** | **Assay** |
| --- | --- | --- | --- |
| N | 2019-nCoV_N1-F | GACCCCAAAATCAGCGAAAT | RT-qPCR |
|  | 2019-nCoV_N1-R | TCTGGTTACTGCCAGTTGAATCTG | RT-qPCR |
|  | 2019-nCoV_N1-P | FAM-ACCCCGCATTACGTTTGGTGGACC-BHQ1 | RT-qPCR |
|  | 2019-nCoV_N2-F | TTACAAACATTGGCCGCAAA | RT-qPCR |
|  | 2019-nCoV_N2-R | GCGCGACATTCCGAAGAA | RT-qPCR |
|  | 2019-nCoV_N2-P | FAM-ACAATTTGCCCCCAGCGCTTCAG-BHQ1 | RT-qPCR |

^a^ Primers were designed by CDC-USA.

**Table S2.** Positive samples for SARS-CoV-2 RNA on public touched surfaces at different locations in Recife, Pernambuco state, Brazil.

| **Location** | **Sample ID*** | **Surface** | **Specific location** | **Type of material** | **Cq value (N1)** |
| --- | --- | --- | --- | --- | --- |
| **Health care units** | 01 | Standing rest bar | Ambulatory entrance | Metal | 37.8 |
|  | 08 | Handle | Hospital access | Metal | 36.2 |
|  | 13 | Doorbell | Hospital access | Plastic | 36.4 |
|  | 14 | Iron grades | Principal entrance | Metal | 38.2 |
|  | 24 | Bench | Principal entrance | Wood | 37.3 |
|  | 36 | Bench | Hospital access | Wood | 36.3 |
|  | 39 | Light switch | Toilet | Plastic | 36.3 |
|  | 43 | Wall | Hospital access | Rock | 35.6 |
|  | 44 | Wall | Hospital access | Rock | 35.5 |
|  | 45 | Handrail | Hospital access | Metal | 37.4 |
|  | 46 | Handrail | Hospital access | Metal | 36.6 |
|  | 47 | Handrail | Hospital access | Metal | 34.8 |
|  | 48 | Handrail | Hospital access | Metal | 36.0 |
|  | 49 | Wall | Hospital access | Rock | 36.2 |
|  | 51 | Phone | Public phone | Plastic | 38.1 |
|  | 52 | Handrail | Hospital access | Rock | 34.8 |
|  | 53 | Trafic light button | Traffic light button | Plastic | 34.8 |
|  | 54 | Trafic light button | Traffic light button | Plastic | 35.2 |
|  | 55 | Trafic light pole | Traffic light button | Metal | 35.3 |
|  | 57 | Trafic light button box | Traffic light button | Metal | 36.6 |
|  | 59 | Park bench | Bus stop | Concrete | 34.4 |
|  | 60 | Bus stop bench | Bus stop | Metal | 36.7 |
|  | 61 | Bus stop ceiling | Bus stop | Plastic | 35.0 |
|  | 62 | Trash box | Bus stop | Plastic | 35.0 |
|  | 63 | Road sign pole | Resting area | Metal | 35.8 |
|  | 67 | Handrail | Resting area | Metal | 37.5 |
| **Transport terminals** | 85 | Faucet | Toilet | Plastic | 34.7 |
|  | 86 | Wall | Toilet | Rock | 33.6 |
|  | 87 | Bench | Bench | Wood | 37.6 |
|  | 88 | Handrail | Handrail | Metal | 36.8 |
|  | 89 | Toilet seat | Toilet | Other (Ceramic) | 35.4 |
|  | 90 | Column | Bus terminal access | Concrete | 35.9 |
|  | 91 | Faucet | Faucet | Metal | 32.2 |
|  | 92 | Bench | Bench | Wood | 35.2 |
|  | 93 | Handrail | Handrail | Metal | 34.1 |
|  | 94 | Faucet | Toilet | Plastic | 34.1 |
|  | 95 | Handrail | Handrail | Metal | 36.7 |
|  | 97 | Bench | Bench | Plastic | 34.9 |
|  | 98 | Handrail | Handrail | Metal | 35.9 |
|  | 99 | ATM button | ATM | Metal | 36.0 |
|  | 100 | Biometrics sensors | ATM | Plastic | 34.4 |
|  | 101 | Box | ATM | Plastic | 35.4 |
|  | 102 | Button | ATM | Metal | 33.3 |
|  | 103 | Biometrics sensors | ATM | Plastic | 35.0 |
|  | 104 | Walls | Bus stop | Metal | 35.4 |
|  | 105 | Protection grid | Bus stop | Metal | 36.0 |
|  | 106 | Button | Ticket machine | Other (Rubber) | 37.6 |
|  | 107 | Screen | Ticket machine | Glass | 33.6 |
|  | 108 | Handrail | Handrail | Metal | 34.1 |
|  | 109 | Wall | Toilet | Rock | 33.6 |
|  | 110 | Bench | Bench | Concrete | 33.7 |
|  | 111 | Roulette | Bus terminal exit | Metal | 33.7 |
|  | 112 | Column | Bus terminal access | Concrete | 33.7 |
|  | 113 | Wall | Subway station access | Rock | 36.5 |
|  | 114 | Handrail | Handrail | Metal | 35.4 |
|  | 116 | Biometrics sensors | ATM | Plastic | 36.2 |
|  | 117 | Faucet | Faucet | Metal | 36.7 |
|  | 120 | Handrail | Handrail | Metal | 36.4 |
|  | 122 | Column | Bus terminal access | Concrete | 36.7 |
|  | 123 | Pipe | Bus terminal access | Metal | 35.3 |
|  | 124 | Protection grid | Bus terminal access | Metal | 36.7 |
|  | 125 | Counter | Counter | Rock | 37.6 |
|  | 127 | Biometrics sensors | ATM | Plastic | 36.4 |
|  | 133 | Bench | Bench | Wood | 37.3 |
|  | 134 | Grid | Subway station access | Metal | 35.4 |
|  | 138 | Protection grid | Bus terminal access | Metal | 38.7 |
|  | 139 | Colunm | Bus terminal access | Concrete | 36.4 |
|  | 141 | Handrail | Handrail | Metal | 36.3 |
|  | 143 | Screen | Ticket machine | Glass | 36.7 |
|  | 144 | Bench | Bench | Wood | 34.6 |
|  | 155 | Keyboard | ATM | Metal | 36.4 |
|  | 156 | Biometrics sensors | ATM | Plastic | 37.3 |
|  | 162 | Handrail | Handrail | Metal | 31.1 |
| **Public parks** | 193 | Toy | Playground | Wood | 37.7 |
|  | 197 | Floor | Recreation area | Concrete | 37.1 |
|  | 205 | Faucet | Toilet | Metal | 37.3 |
|  | 217 | Handrail | Outdoor gym | Metal | 37.3 |
|  | 221 | Bench | Playground | Wood | 37.8 |
|  | 229 | Slide | Playground | Metal | 37.3 |
|  | 231 | Barbell | Outdoor gym | Metal | 37.2 |
|  | 237 | Swing | Playground | Metal | 37.3 |
|  | 242 | Wall | Recreation area | Concrete | 36.3 |
|  | 249 | Tree trunk | Recreation area | Wood | 36.2 |
|  | 281 | Bench | Recreation area | Wood | 37.4 |
|  | 288 | Handrail | Handrail | Metal | 37.6 |
|  | 305 | Faucet | Toilet | Plastic | 39.7 |
|  | 311 | Barbell | Playground | Metal | 37.4 |
| **Public markets** | 319 | Faucet | Toilet | Metal | 37.5 |
|  | 320 | Door | Toilet | Wood | 37.9 |
|  | 360 | Handrail | Principal entrance | Metal | 36.9 |
|  | 386 | Grade | Toilet | Metal | 38.1 |
| **Beach areas** | 177 | Tap | Toilet | Metal | 36.1 |
|  | 180 | Handrail | Resting area | Wood | 37.0 |
|  | 181 | Wall | Toilet | Rock | 37.4 |
|  | 188 | Bench | Bench | Rock | 37.9 |
| **Supply center** | 258 | Handrail | Handrail | Metal | 38.0 |
|  | 261 | Faucet | Toilet | Plastic | 38.7 |

* Samples were numbered 1 to 400. Numbers shown are the ID of the sample tested positive by RT-qPCR.

**Table S3.** Viability of SARS-CoV-2 positive surface samples using the Vero CCL-81 linage.

| **ID sample** | **Material** | **Swab**  **(Cq value)** | **P3 – 0h**  **(Cq value)** | **P3 – 72h**  **(Cq value)** | **Interpretation** |
| --- | --- | --- | --- | --- | --- |
| 86 | Rock | 33.6 | ND | ND | Non-culturable |
| 91 | Metal | 32.2 | ND | ND | Non-culturable |
| 102 | Metal | 33.2 | ND | ND | Non-culturable |
| 107 | Glass | 33.6 | ND | ND | Non-culturable |
| 109 | Rock | 33.6 | ND | ND | Non-culturable |
| 110 | Concrete | 33.7 | ND | ND | Non-culturable |
| 111 | Metal | 33.7 | ND | ND | Non-culturable |
| 112 | Concrete | 33.7 | ND | ND | Non-culturable |
| 162 | Metal | 31.0 | ND | ND | Non-culturable |

P3: Passage 3

**Table S4.** Evaluation of safety procedure protocol implementation against COVID-19 at collection areas (n=19).

| Variables | Frequency | Percent (%) |
| --- | --- | --- |
| Availability of 70% alcohol at the entrance |  |  |
| Yes | 5 | 26.3 |
| No | 14 | 73.7 |
| Availability of faucets and soap for handwashing |  |  |
| Yes | 8 | 42.1 |
| No | 11 | 57.9 |
| Temperature measurement at the entrance location |  |  |
| Yes | 3 | 15.8 |
| No | 16 | 84.2 |
| Availability of informative material on preventive measures against COVID-19 |  |  |
| Yes | 8 | 42.1 |
| No | 11 | 57.9 |
| People wearing mask |  |  |
| Yes | 18 | 94.7 |
| No | 1 | 5.3 |
| Social distancing^a^ |  |  |
| Yes | 5 | 26.3 |
| No | 14 | 73.7 |
| Control of the number of persons accessing the area |  |  |
| Yes | 1 | 5.3 |
| No | 18 | 94.7 |

^a^ considering 2 m
